# Comparative Genomics of *Xanthomonas citri* pv. *citri* A* Pathotype Reveals Three Distinct Clades with Varying Plasmid Distribution

**DOI:** 10.3390/microorganisms8121947

**Published:** 2020-12-08

**Authors:** John Webster, Daniel Bogema, Toni A. Chapman

**Affiliations:** NSW Department of Primary Industries, Elizabeth Macarthur Agricultural Institute PMB 4008, Narellan, NSW 2570, Australia; Daniel.bogema@dpi.nsw.gov.au (D.B.); Toni.chapman@dpi.nsw.gov.au (T.A.C.)

**Keywords:** *Xanthomonas citri*, A* pathotype, plasmid, complete genome, pan-genome, phylogenomics

## Abstract

Citrus bacterial canker (CBC) is an important disease of citrus cultivars worldwide that causes blister-like lesions on host plants and leads to more severe symptoms such as plant defoliation and premature fruit drop. The causative agent, *Xanthomonas citri* pv. *citri*, exists as three pathotypes—A, A*, and A^w^—which differ in their host range and elicited host response. To date, comparative analyses have been hampered by the lack of closed genomes for the A* pathotype. In this study, we sequenced and assembled six CBC isolates of pathotype A* using second- and third-generation sequencing technologies to produce complete, closed assemblies. Analysis of these genomes and reference A, A*, and A^w^ sequences revealed genetic groups within the A* pathotype. Investigation of accessory genomes revealed virulence factors, including type IV secretion systems and heavy metal resistance genes, differentiating the genetic groups. Genomic comparisons of closed genome assemblies also provided plasmid distribution information for the three genetic groups of A*. The genomes presented here complement existing closed genomes of A and A^w^ pathotypes that are publicly available and open opportunities to investigate the evolution of *X. citri* pv. *citri* and the virulence factors that contribute to this serious pathogen.

## 1. Introduction

Citrus bacterial canker (CBC), a disease caused by the pathogen *Xanthomonas citri* pv. *citri*, is a serious phytopathogen of a wide variety of citrus cultivars. An infection of CBC causes the development of unsightly, blister-like lesions on the host plant’s leaves, stems, and fruits [1]. In advanced infections, canker causes plant defoliation and can lead to premature fruit drop. These effects cause considerable economic loss internationally, with extra implications to regions without CBC and their market access should exotic incursions of CBC arise. Examples of this include the recent incursions into Australia in 2004 and 2018, with the 2004 eradication having been estimated by the Australian Bureau of Agricultural and Resource Economics to have provided a net benefit of AUD 70 million to Queensland alone had eradication not occurred [2]. In 2016-2017, Australia’s citrus industry was valued at AUD 724.4 million, with a large proportion (AUD 462 million) being for exports, and in 2019, Australian citrus export exceed AUD 541 million [3,4].

The host range for CBC is broad and encompasses most species of citrus (Queensland Biosecurity Regulation 2016, Schedule 7A, section 57B [5]) and other species of Rutaceae [6]. However, three pathotypes for *X. citri* pv. *citri*—A, A*, and A^w^—have been assigned on the basis of host specificity and the defensive responses enacted by those host species. Of these three pathotypes, pathotype A infects the broadest host range, infecting most species of *Citrus*. Pathotypes A* and A^w^ have a comparatively narrower host range, infecting Key lime (*Citrus aurantifolia*) and alemow (*Citrus macrophylla*), and either not producing symptoms on grapefruit (A*) or eliciting a hypersensitive response on grapefruit (A^w^) [7,8,9]. Previously, the genetic diversity of *X. citri* pv. *citri* was explored using next-generation sequencing (NGS) [10,11,12] to elucidate the evolutionary history of *X. citri* pv. *citri*’s three pathotypes. The relative position of the pathotypes in the *X. citri* pv. *citri* phylogeny has been inconsistent with multiple studies observing different evolution of the A, A*, and A^w^ pathotypes. Patané et al. (2019) attribute these inconsistencies in lineages of *X. citri* pv. *citri* to be due to fast diversification, causing a “blurring effect” of the phylogeny [13]. Additionally, Patané et al. (2019) identified a clade of isolates that they observed in their analyses, as well as the analyses of Gordon et al. [10] and Pruvost et al. [14], which they named A2. The authors noted, however, that there was no phenotypical difference in the A2 clade and A clade of which they knew [13].

Like other xanthomonads and most other Gram-negative bacteria, *Xanthomonas citri* pv. *citri* use type 3 secretion systems (T3SS) to deliver key pathogenicity effectors to host cells [15,16]. Of these type 3 effectors (T3Es), transcription activator-like effectors (TALE) are one of the largest effector families that are found primarily in xanthomonads [17,18]. These TALE are translocated to the host cell via a hollow pilin and modulate host cell responses by binding and acting as a transcriptional activator to host gene promoter regions, expression of which facilitates infection [17,19]. An example of this, and one of the most well studied effectors, is the TALE PthA, which is responsible for symptoms through the induction of the canker susceptibility gene CsLOB1 [20]. The PthA effector is represented in all three pathotypes of X. *citri* pv. *citri* and has been shown to be essential for pustule formation by induction of the CsLOB1 gene, facilitating cell hypertrophy and hyperplasia and leading to the characteristic canker pustules [20,21,22,23]. Patané et al. (2019) investigated a set of 120 pathogenicity-associated genes in X. *citri* pv. *citri* and also identified 18 effectors (hpaA, xopAD, xopAK, xopAP, xopE1, xopE2, xopF1, xopF2, xopK, xopL, xopM, xopN, xopQ, xopR, xopS, xopV, xopX, and xopZ1). In addition to T3SS, *X. citri* pv. *citri* has also been documented to contain type IV secretion systems (T4SS) [24]. Type IV secretion systems have been characterised in a range of Gram-positive and -negative bacteria and act as translocators of proteins and DNA to host cells and also play a role in conjugation [25,26]. In X. *citri* pv. *citri*, T4SS have recently been identified to be produced at a basal level and regulated by the conserved global regulator CsrA [27]. It was observed that *X. citri* pv. *citri* utilised T4SS to transfer a cocktail of antibacterial effector proteins into other Gram-negative bacteria, leading to rapid lysis of the targeted cells and providing itself a fitness benefit against competing bacteria [28,29].

This study aimed to investigate the A* pathotype by creating hybrid assemblies of six *X. citri* pv. *citri* isolates using a combination of Illumina and Oxford Nanopore reads to produce closed genome sequences. Accessory genes are often harboured in mobile genetic elements such as transposons and plasmids and are important drivers in niche survival and pathogenesis [30,31] as well as emergence of plant pathogens. Bacterial pathogens can rapidly emerge via the acquisition of genes through horizontal gene transfer, leading to novel pathogen genotypes that may become more virulent or show increased fitness in other host organisms, resulting in host jumps [32,33,34]. An example is the discovery of copper resistance in *X. citri* pv. *citri* isolates from both Argentina and Florida in which resistance genes have been found on plasmids [35]. Closed assemblies are useful for investigating these mobile genetic elements as well as the genomic localisation of genes, pangenomes, and plasmid structure. In turn, this helps in understanding virulence and evolution, and is useful for tracking of incursion isolates. Short read assemblies are difficult to assemble completely due to repeat regions that are longer than sequencing read lengths. These repeat sequences can then generate “breakpoints” at regions of importance, such as accessory genes that contain repeat elements, for example xanthomonas TALE [36,37]. This work presents, for the first time, complete genomes of isolates from the A* pathotype and investigates accessory genes present within three genetic groups of *Xanthomonas citri* pv. *citri* A*.

## 2. Materials and Methods

### 2.1. Isolates

Isolates were obtained from the New South Wales (NSW) Department of Primary Industries, Biosecurity Collections, Plant Pathology and Mycology herbarium (Orange, Australia), which are detailed in Table 1.

### 2.2. DNA Extraction

Extraction of high molecular weight DNA for genome sequencing on an Oxford Nanopore Technology (Oxford, UK) MinION was performed using the Genomic Tip 20/G kit (Qiagen) according to the manufacturer’s protocol. Isolates were grown on nutrient agar plates at 25 °C until sufficient growth was observed (≈3 days). Bacterial biomass was transferred to a 1.5 mL microfuge tube and rinsed twice with 1 mL of phosphate-buffered solution before DNA extraction was performed. 

Extraction of DNA for Illumina library preparation was performed using a DNeasy Blood and Tissue Kit (Qiagen) according to the manufacturer’s protocol.

### 2.3. Library Preparation and Sequencing with Illumina and Minion

Oxford Nanopore sequencing was performed PCR-free with barcoded sequences using the Ligation Sequencing Kit (Oxford Nanopore) with the Native Barcoding expansion and loaded onto a MinION R9.4.1 flow cell. Libraries were prepared according to the manufacturer’s protocol with the DNA fragmentation step skipped to ensure long reads, and long fragment selection was performed as part of the ligation kit protocol, prior to library loading. Flow cell QC showed 1559 active pores initially, and sequencing, through MinKNOW, was run for 48 h. Live basecalling was performed with MinKNOW and resulting fastq files were used for hybrid assembly.

Isolates were short-read sequenced by the Australian Centre for Genomic Epidemiological Microbiology (https://www.ausgem.net/) using an Illumina HiSeq, as described previously [38].

### 2.4. Data Processing, Assembly, and Sequence Analysis

Long reads from ONT MinION sequencing were demultiplexed and filtered using Porechop v0.2.4 [39] with the “--discard_middle” flag to remove any reads with internal barcodes. Further filtering was performed with FiltLong v0.2.0 [40] to remove reads shorter than 2000 bp while keeping the top 90% of the remaining reads.

Hybrid assembly of quality filtered Illumina and ONT reads was performed using Unicycler v0.4.8 [41] with bridging mode set to normal. Flye v2.5-g315122d [42] assemblies with Pilon v1.23 [43] polishing were also produced. This was achieved by firstly assembling ONT reads with Flye using an estimated genome size of 5 Mb. An index of the ONT assembly was created and Illumina forward and reverse reads were mapped to the assembly using bowtie2 v2.3.4.1 [44] and the resulting SAM file converted to BAM for sorting and indexing with SAMtools v1.7 [45]. One round of Pilon polishing was then performed using the ONT Flye assembly and BAM files. All tasks were automated with a Snakemake v5.5.0 [46] workflow. Manual completion of small tangled plasmids was achieved using Bandage v0.8.1 [47] with the Basic Local Alignment Search Tool (BLAST) algorithm to compare long reads against tangled contigs to find reads with supporting evidence for the separation of assembly graph nodes. Assemblies from Unicycler and Flye were compared using a mauve v2.3.1 plugin in Geneious v9.1.8 (https://www.geneious.com) to compare chromosome arrangements between assemblies and BUSCO v3.0.2 [48] to assess completion of the assemblies. Completed assemblies were submitted to the National Center for Biotechnology Information (NCBI) under Bioproject PRJNA657081. Average nucleotide identity (ANI) was calculated on genomes sequenced in this study against reference A, A*, and A^w^ genomes using the calculator available at http://enve-omics.ce.gatech.edu/ani/ to confirm the genomes sequenced were X. *citri* pv. *citri*.

### 2.5. Identification of Type 3 Secreted Effectors

All genomes were investigated for 65 T3SE (see Table S2) with TBLASTN. Effectors were considered present if a translated subject nucleotide showed ≥80% percent amino acid identity to the queried T3SE and covered ≥80% of the query length. Custom in-house scripts were used to determine the presence/absence of these effectors from TBLASTN results. Effectors that were below the coverage and identity thresholds or absent in all genomes are not shown.

### 2.6. Phylogenetic Analysis and Pan-Genome

Snippy v4.4.5 [49] was used to produce variant calls for *X. citri* pv. *citri* genomes (Table S1) and to align all polymorphic sites with X. *citri* pv. *citri* UI6 as a reference sequence. Recombination filtering was performed with Gubbins [50] on the core Snippy alignment, and a maximum likelihood phylogenetic tree of single-nucleotide polymorphisms (SNPs) was inferred with RAxML with GTRGAMMA used as the base-substitution and rate heterogeneity model; branch support values were generated using 100 bootstrap replicates. Phylogenies were then imported into the Interactive Tree of Life (iTOL- https://itol.embl.de/) and midpoint-rooted. 

Genome annotation was performed with Prokka v1.14.5 [51], and resulting general feature format files were used for pan-genome analysis. Two pan-genomes were inferred with Roary v3.12.0 [52]. The first pan-genome used the 6 sequenced *X*. *citri* pv. *citri* A* genomes in this study to investigate gene content differences within genetic groups. The second, which also incorporated 32 reference genomes from publicly accessible NCBI *X*. *citri* pv. *citri* genomes with “complete” assembly level, examined the evolution of the pathogen. For both pan-genomes, a gene presence/absence matrix was produced from Roary. A plot produced with roary_plots.py incorporating the SNP phylogeny was also created for the pan-genome that included the reference isolates. The distribution of genes amongst the various combinations of the A* genomes in the A*-only pan-genome were visualised with UpSetR [53].

Panaroo v1.2.3 [54] was used to determine the number of structural recombination events between all closed genomes used in this study by creating 3 separate Panaroo graphs (for the 3 separate pathotypes) and merging the graphs with Panaroo-merge.

## 3. Results

### 3.1. Assembly Completion

In most cases, assembly using Unicycler was able to resolve plasmid sequences more consistently than assembly with Flye and Pilon polishing, except for DAR73910, where plasmids were assembled completely with Flye. To detect any misassemblies, chromosomes from both Flye and Unicycler assemblers and a reference *X. citri* pv. *citri* (UI6- GCA_000961175.1) were compared using Mauve, which showed that chromosomes from both the Flye and Unicycler assemblers were structurally congruent. To assess assembly completion, BUSCO (Benchmarking Universal Single-Copy Orthologs), with the gammaproteobacteria_odb9 OrthoDB v9 database, was run on all Flye and Unicycler assemblies, which showed that Unicycler assemblies contained 95.8 to 96.4% complete BUSCOs of the 452 BUSCO groups searched. Additionally, all Unicycler genomes were only missing 11 BUSCOs (2.4%) and contained between 5 and 7 (1.1 to 1.5%) fragmented BUSCOs. Flye assemblies polished with Pilon, however, contained a lower percentage of complete BUSCOs (84.5 to 94%) and a higher proportion of fragmented (2.8 to 10.1%) and missing (3.1 to 6.4%) BUSCOs. As such, Unicycler assemblies were used for further analyses, except for DAR73910, which showed better assembly with Flye on the basis of the proportion of BUSCOs recovered and the complete assembly of DAR73910 plasmids.

### 3.2. General Features of Xanthomonas citri *pv.* citri A* Genomes

Six *X. citri* pv. *citri* A* isolates were genome sequenced and assembled de novo into complete closed genomes in this study; their general features are shown in Table 2. Average nucleotide identity (ANI) was performed, initially confirming that all genomes were closely related (99.9145 to 99.9948%). All genome assemblies had circular chromosomal contigs of sizes between 5.17 and 5.22 Mb and contained between four and five circular, extrachromosomal contigs of sizes 24 to 220 kb. A mean G+C content of 64.59 to 64.73% was observed for all isolates, matching G+C averages of other published *X. citri* pv. *citri* genomes. The number of coding sequences (CDS) found amongst the genomes, on the basis of the Prokka annotation pipeline, ranged from 4390 (DAR72029) to 4851 (DAR73910). 

### 3.3. Placement of A* Strains amongst X. citri *pv.* citri Population

**Figure 1:**
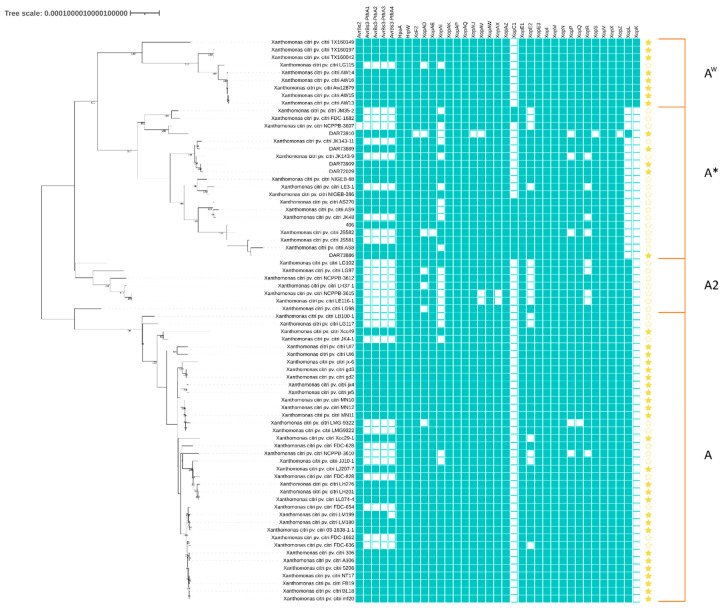
Maximum likelihood phylogeny with 100 bootstrap replicates, midpoint-rooted of a recombination filtered alignment of 4,246,524 bp core Snippy alignment across 74 *Xanthomonas citri* pv. *citri* genomes. Presence/absence of type III secreted effectors also shown (green) and complete level genomes are represented with a filled star (yellow). Tree scale is represented as number of substitutions per site.

A Snippy core genome alignment of 4,246,524 bp was filtered for recombination and phylogeny inferred for the six genomes presented here, along with 68 publicly accessible genomes of *X. citri* pv. *citri* from NCBI at varying levels of completion. Phylogenetic analysis revealed three large monophyletic groupings that corresponded to the *X. citri* pv. *citri* pathotypes A, A^w^, and A* (Figure 1), with pathotype A2 also present. Isolates sequenced in this study were separated into three distinct genetic groups within the A* clade. These genetic groups were labelled A*^a^ (containing isolates DAR84832 and DAR73886), A*^b^ (DAR72029, DAR73909, and DAR73889), and A*^c^ (DAR73910) for the purpose of this manuscript. 

### 3.4. Pan-Genome Analysis

Both pan-genomes of *X. citri* pv. *citri* were inferred with Roary by performing an all-against-all BLASTP with a minimum percentage identity of 95% for all genomes in this study, in addition to 32 reference *X*. *citri* pv. *citri* isolates sourced from NCBI with assemblies at complete level. Results of the SNP phylogeny showed that there were no genomes of complete level that had clustered as an A* pathotype; as such, the genomes sequenced in this study were the only A* representatives. Complete level genomes were used for pan-genome creation to prevent underestimation of the core genome and overestimation of the accessory genome due to partial or missing annotations of genes on contig ends. Using complete genomes also allows for identification of plasmid vs. chromosomally located pan-genome genes. 

**Figure 2:**
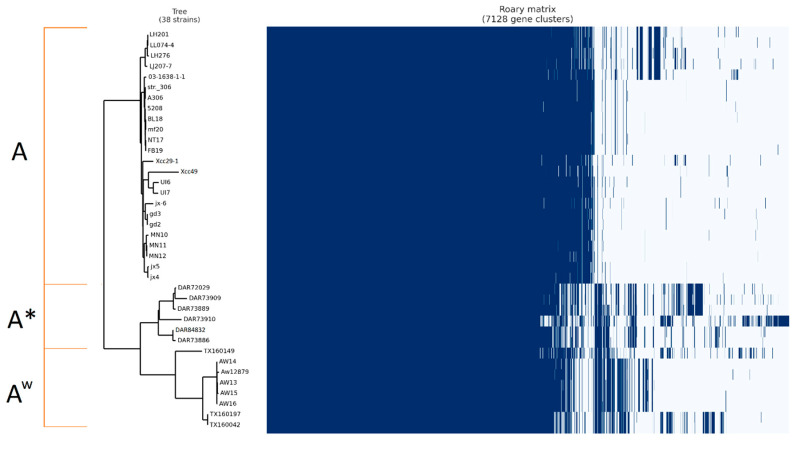
Roary pan-genome matrix of *Xanthomonas citri* pv. *citri* A, A*, and Aw pathotypes of sequenced and reference isolates. Figure produced using presence/absence pangenome matrix and Snippy core alignment filtered with gubbins. Blue bars represent genes present in the genome.

The pan-genome of the 6 isolates in this study and 32 NCBI reference isolates contained 5621 gene clusters, of which 4202 (74.75%) made up the core genome, and the accessory genome contained 1419 genes (Figure 2). As with the SNP tree in Figure 1, isolates are shown to have grouped into three distinct clades, representing A*, A^w^, and A pathotypes. 

**Figure 3:**
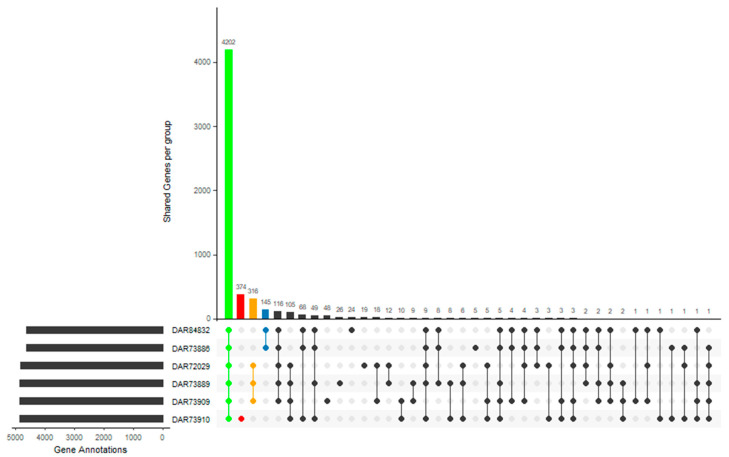
Visualisation of intersecting gene sets of the genomes sequenced in this study. Coloured linked dots represent isolate groupings for the core genome (green) A*^c^ (red), A*^b^ (orange), and A*^a^ (blue) and the number of genes in that grouping is represented by the bar plots above.

The pan-genome of the A* pathotype showed the presence of genes that were unique to each of the three genetic groups (A*^a^, A*^b^, and A*^c^) (Figure 3). While 145, 316, and 374 genes were observed to be unique to A*^a^, A*^b^, and A*^c^ genetic groups, respectively, only 42, 72, and 87 of these genes were putatively assigned functions. Genomes from the A*^b^ genetic group contained more plasmids and plasmids of larger sizes, and as such had more annotated genes (4789 to 4845) compared to the slightly smaller genomes of A*^a^ (4592 to 4616). Each genetic group was also similar in terms of plasmid profiles, with plasmids of sizes of 61, 47, 28, and 25 kb in both A*^a^ isolates and plasmids of sizes 220, 56, 42, 27, and 24 kb in all three A*^b^ isolates. Plasmids of sizes 116, 38, and 28 kb in A*^c^ were also different to those found in the other two genetic groups. Plasmids were designated an ID on the basis of their size, or, in the case of pXAC64 and plasmid pF, BLAST searches with high identity to previously identified plasmids. There was also a total of 2318 structural recombination events, as calculated by Panaroo.

All genomes contained a chromosomally located T4SS operon; however, in A*^a^, unique homologues for T4SS were located on a 61 kb plasmid pXAC64, and the components of this T4SS exhibited different organisation and sequence identity to those in the chromosome. This represented 15 of 145 unique genes in A*^a^. In contrast, only 1 of 316 genes unique to A*^b^ were associated with a T4SS, with this genetic group instead possessing a high number of genes involved in heavy metal resistance for cobalt, zinc, cadmium, and copper. In A*^c^, however, only one unique gene, *czcA*, for the cobalt–zinc–cadmium resistance protein was annotated while none were annotated with a T4SS. Accessory genes for T4SS in A*^a^ and heavy metal resistance in A*^b^ were distributed amongst plasmids in these two genetic groups. 

In addition to T4SS and heavy metal resistance, three genes for the production of a putative endoribonuclease, *mazF*, were present in the A*^a^ genetic group. These genes were present in combination with a complementary antitoxin gene, *mazE*, forming a toxin–antitoxin (TA) system on plasmids pXAS28 and pXAC64. A *yafQ*-*dinJ* TA system was also uniquely present in the A*^a^ genetic group on the pXAS28 plasmid. Another TA system toxin gene, *fitB*, was present in all genetic groups and located chromosomally. However, a second copy of *fitB* was also found on the pXAS28 plasmid within the A*^b^ genetic group whose sequence is unique to that group. A full list of these genes is available in Table S3.

### 3.5. Type 3 Secreted Effectors

A total of 65 type 3 secreted effectors (T3SE) were investigated in the six A* isolates sequenced in this paper, as well as all *X*. *citri* pv. *citri* references from NCBI (Figure 1). Of the 65 T3SE investigated, the 6 A* isolates examined in this study contained 26 to 33 different T3SE that met the search criteria. Most of these T3SE identified through TBLASTN were chromosomally located in the A* isolates and varied in amino acid sequence between the three genetic groups for non-TALE TS3E: HpaA, XopAD, XopAI, XopAU, XopS, XopX, XopE2, and XopAE (99.8%, 99.9%, 99.8%, 99.8%, 99.8%, 99.9%, 98.9%, and 99.9% pairwise identity respectively). Effectors XopQ and XopE2 also showed truncated TBLASTN results in DAR73910 (coverage of 420/464 and 356/401 positions, respectively). A second copy of XopE2 was also found on the pXAS38 plasmid in the A*^c^ isolate DAR73910. Multiple copies of TALE (PthA1-4) were located on the pXAS47 plasmid in A*^a^, the pXAS42 plasmid in A*^b^, and the pXAS38 plasmid in A*^c^. TALE proteins consisted of central regions of polymorphic repeats ranging between 14 and 20 repeats of 33-34 amino acid residues. Repeat-variable diresidues were observable at positions 12 and 13 with the following proportions of NG: 0.2165, NS: 0.0563, NI: 0.2254, HD: 0.3574, and N*: 0.1444 across all TALE repeats. Repeat variable positions were also noted at positions 3, 4, and 11. Of all the TALE-filtered TBLASTN results, only full-length sequences were found in DAR73886 for PthA1-3, DAR84832 for PthA1-4, DAR72029 for PthA2-4, DAR73909 for PthA2-3, DAR73889 for PthA1-4, and DAR73910 for PthA1-4 (Table 3). Of note, TALE were often not identified in incomplete reference genomes, likely due to issues assembling short read assemblies at *tale* repetitive regions. The three pathotypes—A, A*, and A^w^—differed in distribution of T3SE, with the XopK effector only found in A^w^ isolates and XopL found in A and A^w^ but not A*. However, the A*^c^ isolate, DAR73910, contained an XopL effector. All A* isolates (except DAR73910) resulted in two TBLASTN hits with >99% ID to the reference XopL that each had <80% coverage (200 and 297 of 497 amino acid residues), but together accounted for the complete length of the reference. These two TBLASTN hits represented separate open reading frames and were separated by a 1 bp insertion.

## 4. Discussion

The isolates studied in this paper represent the first complete assembly of A* genomes, and the analyses presented here examine three genetic groups of the A* pathotype. A core genome alignment of 4,246,524 (Figure 1) positions filtered for recombination showed four monophyletic groupings of *X. citri* pv. *citri* pathotypes, A, A*, A^w^, and A2. This topology of pathotypes with [A* + A^w^] sharing a branch to the exclusion of A was consistent with that seen by Zhang et al. (2015), who also observed [A* + A^w^]. However, this topology does not match Patané et al.’s findings (2019), wherein the authors observed [A + A^w^] pathotypes sharing a branch using core gene and Locally Collinear Block (LCB) alignments. According to Patané et al. (2019), the common topology appears to be that of [A + A^w^]. This topology of [A + A^w^] they overserved matched Gordon et al.’s observations, although this topology often had low support [10]. Patané et al. suggested that the topology observed by Zhang et al. was likely due to the low number of genomes used, as well as significant recombination within *X. citri* pv. *Citri*, complicating phylogenetic reconstruction [13]. However, Cubero and Graham (2002) also noted, using BOX and ERIC PCR, that A* and A^w^ strains in Florida were related and suggested a common origin in Southwest Asia [55]. Genome-wide homologous recombination has been observed in a number of *Xanthomonas* spp. [56,57] and is likely the complicating factor involved in accurately inferring phylogenetic relationships, as homologous recombination is not indicative of vertical evolution and can mask the signal of temporal evolution [58,59]. In this study, a large number of genomes were used to generate recombination-filtered SNP alignments, resulting in a topology of [A* + A^w^] sister groups to the exclusion of the A pathotype. While the results from Patané et al.’s and Gordon et al.’s works could not be replicated with multiple recombination and tree building methods here (data not shown), the genetic group grouping remained consistent.

The A* pan-genome showed the presence of several genes that are unique to the genetic groups and are plasmid-located. These unique genes include, but are not limited to, genes for T4SS and heavy metal resistance. Each of the genomes investigated here contained the gene cluster for construction of a VirB/D4 T4SS that was chromosomally located. Type IV secretion systems are ubiquitous in bacteria and archaea, and function as delivery systems for DNA and proteins for a variety of purposes. The production of a cell envelope-traversing protein complex in T4SS facilitates the movement of proteins and protein–DNA complexes through a protein channel to target cells in a contact-dependent manner [60]. Target cells can include eukaryotic hosts where virulence factors have been shown to be translocated via T4SS and bacterial cells where T4SS assist in the horizontal transfer of genetic information, such as plasmids. Genetic group A*^a^ also harboured a 61 kb plasmid that contained additional genes for a T4SS with similar structure to the pXAC64 megaplasmid that has previously been shown to contain a copy of the T4SS [61] and is identified here as pXAC64. This 61 kb plasmid included additional T4SS genes, two copies of the T3SE *pthA*, three toxin/antitoxin systems, toxin zeta, relaxosome proteins, and two insertion elements—ISXc4 and TnXo19. This plasmid is found in both isolates from genetic groups A*^a^ and represents a significant source of virulence factors that are involved in phytopathogenesis and genome plasticity genes. A second plasmid, pXAS28, also contained a third copy of the T4SS which varied in nucleotide identity compared to the T4SS of the pXAC64 plasmid. The presence of divergent copies of various T4SS genes on the chromosome and two plasmids, such as genes VirB5 and VirB2 that are part of the outer-membrane pilin, likely represent the evolution of proteins and the host–pathogen “arms race” at the extracellular interface between *X. citri* pv. *citri* and its hosts [24,60].

Genes for heavy metal resistance for metals such as copper, cobalt, cadmium, and zinc were also present and unique for genetic groups A*^b^. The use of copper-based bactericides is common in the integrated management of canker and, as such, copper resistance has developed in several plant pathogens, including CBC [35]. This resistance has been shown to be conferred by resistance genes located mostly on plasmids, although chromosome-based resistance has also been observed [35]. All isolates studied here showed the presence of copper resistance genes *copA* and *copB*, located chromosomally. In addition to this, isolates from genetic group A*^b^ also possessed *copA* and *copB* homologs as well as a copper chaperone protein *copZ* and copper-transporting ATPase all on their 220 kb plasmid. The presence of copper resistance on the A*^b^ 220 kb plasmid represents a potentially autonomous mobile element of copper resistance that can be quickly transferred between various strains and species of *Xanthomonas* [62]. Such rapid acquisition of plasmid-borne copper resistance has been observed in other plant pathogens such as *Pseudomonas syringae* pv. *actinidae* (Psa) causing kiwifruit canker. At the time of introduction of Psa to New Zealand in 2010, the outbreak isolate was sensitive to copper. However, within 5 years, a quarter of isolations were resistance to copper, cadmium, and arsenic through integrative conjugative elements and plasmids [63]. Copper spray is used in citrus culture as a protective measure against CBC and is applied regularly to ensure that new growth is consistently covered [64,65]. Regular extensive use of copper applies selective pressure to CBC, and as such, copper resistance has emerged [35].

Results from TBLASTN showed the presence of the T3SE, XopL, in all A^w^, A, and A2 isolates as well as in the A* isolate, DAR73910, sequenced in this study. All other A* isolates were not shown by TBLASTN to contain an intact *xopL* sequence with >80% coverage to the reference. However, two hits in separate open reading frames with >99% ID to the reference were observed in all other A* genomes. When combined, these two separate hits covered the entire reference length, although were separated by one base pair that appears to have caused a frameshift mutation, affecting the latter half (297/497) of the amino acids in the XopL effector. This single base pair insertion was observed in all Flye and Unicycler assemblies and was not an artefact of DAR73910 being assembled with Flye. Gordon et al. also observed the presence of nonsynonymous SNPs in *xopL* specific to the A, A^w^, and A* pathotypes; however, only nonsynonymous SNPs were observed in A* in this study. For this reason, Gordon et al. suggested, due to the distribution of *xopL* across the three *X. citri* pv. *citri* pathotypes, that it was unlikely the root cause of host range differences. However, XopL has previously been shown to be required for full virulence of *Xanthomonas campestris* pv. *campestris* on Chinese radish plants, part of the Brassicaceae family [66], and as it was only observed in A* pathotypes in this study, the role of XopL in host range differences should not be discounted. Recognition of various pathogen-associated molecular patterns (PAMPS) is an important first step of PAMP-triggered Immunity (PTI) [67], and to be successful, pathogens need to be able to subvert these defences [68]. In previous studies, XopL has been shown to significantly inhibit the defence response of *Arabidopsis thaliana* protoplasts to two PAMPS; the bacterial flagellin conserved epitope (flg22) and to an 18 amino acid peptide of the elongation factor thermo unstable protein (elf18) in bacteria [69]. The ability of *X. citri* pv. *citri* to subvert the PTI in response to bacterial flagellin is likely an important step in the pathogenic process, as canker lesions and biofilm production has previously been linked to the flagellar apparatus, with *flgE* mutants producing statistically significantly less lesions than wild-type strains [70]. The lack of an intact XopL effector may help account for the narrower range of host virulence observed in A*. Patané et al. (2019) also screened 120 genes against a range of *X. citri* pv. *citri* genomes, and their results differed slightly from those presented in this manuscript. In their results, XopAE (several), AvrBs2 (NCPPB3607), XopAU (DAR73910), and XopP (JS582) were found to be present in all *X. citri* pv. *citri* pathotypes, whereas XopAE was missing in JS582, AvrBs2 was missing in NCPP3607, and XopAU was missing in the newly sequenced DAR73910 in this study, whereas XopE3 was missing in Xcc29 but was found to be present in this study. Other effectors, such as XopE2, XopL, and XopK, were found in all isolates in the study, but were absent in large numbers. These discrepancies appear to have been caused by different filtering criteria used in TBLASTN searches, with Patané et al. employing an e-value cutoff of ≤1 × 10^−50^, while in this study, a coverage and percent identity of 80% was used. Both methods are justifiable, with Patané et al.’s threshold allowing a greater number of incomplete sequences (e.g., as seen with the two open reading frames of XopL in this study; neither reached 80% coverage, but each separate hit had an e-value of 0) to pass through the filtering criteria. This resulted in a seemingly wider coverage of T3Es in the genomes. The stricter methods employed here helped elucidate differences in T3E sequence between the pathotypes, such as XopL in A and A^w^ but not A* pathotypes, and XopK in A^w^ but not A* and A pathotypes. Similar results for T3Es investigated in both this study and Hajri et al.’s study were found, with XopB, XopD, XopJ, and XopO being absent from *X. citri* pv. *citri* [71]. While XopA and XopC were found in two and one isolates, respectively, in Hajri et al.’s study, none were observed in this study and were likely due to the methods of identification (PCR vs TBLASTN).

## 5. Conclusions

This study aimed to provide the research community with complete level genomes for six isolates of *X. citri* pv. *citri* by sequencing with both Illumina and ONT technologies and producing hybrid assemblies. Producing complete, circular genomes is important to achieve an understanding of how plant pathogens function. Short read assemblies often break at points of sequence repetition and, as evident from TBLASTN results in this manuscript, can lead to the absence of accessory genes in the final assembly. These results also provide evidence of pathogenesis and fitness factors on mobile elements within the A* genome and are useful for understanding how the emergence of disease and resistance occur. Pan genomes of the sequenced A* isolates showed the presence of a range of virulence and fitness factors, such as T4SS and metal resistance genes, likely driven by the use of antimicrobial agents to control CBC. Pangenomes also show that *X. citri* pv. *citri* A* accessory genomes are largely plasmid-driven. These genomes represent the first circular A* chromosome and plasmid sequences and are important for future understanding of the evolutionary history of CBC. Using these genomes and publicly available genomes, an alignment of recombination filtered SNPs showed the presence of three large monophyletic groupings of the three major pathotypes of *X. citri* pv. citri, with [A* + A^w^] as sister groups to the exclusion of A. While three genetic groups of the A* pathotype were investigated here, there appears to be further delineations that are worth including in future work. Australia has in recent years experienced incursions of exotic CBC and biosecurity response efforts have thus far been successful in the eradication of the disease. Genomics has played a vital role in the identification and tracking of incursion isolates and is generally a valuable tool for emergency responses; moreover, having complete reference sequences increases capacity for diagnostics.

## Figures and Tables

**Table 1:** Isolates genome sequenced in this study.

Isolate	Location	Year
DAR73910	International intercept from India at Sydney Airport	2000
DAR73909	Thailand	2000
DAR73886	Iran	Unknown
DAR73889	International intercept from Thailand at Sydney Airport	Unknown
DAR72029	International intercept from Singapore at Sydney Airport	1997
DAR84832	Southwest Asia	Unknown

**Table 2:** General features of chromosomal and plasmid DNA in assembled *X. citri* pv. *citri* A*.

	Size (bp)	GC Content	Total CDS	tRNA	rRNA
DAR84832					
Chromosome	5,223,652	64.7	4429	58	6
*Plasmid 1 (pXAC64)*	61,273	61.4	67	0	0
*Plasmid 2 (pXAS47)*	47,525	60.6	54	0	0
*Plasmid 3 (pXAS28)*	28,106	60.5	35	0	0
*Plasmid 4 (pXAS25)*	25,259	63.4	31	0	0
DAR73886					
Chromosome	5,208,945	64.7	4407	58	6
*Plasmid 1 (pXAC64)*	61,267	61.4	67	0	0
*Plasmid 2 (pXAS47)*	47,520	60.6	52	0	0
*Plasmid 3 (pXAS28)*	28,110	60.5	35	0	0
*Plasmid 4 (pXAS25)*	25,259	63.4	31	0	0
DAR73909					
Chromosome	5,178,469	64.9	4424	59	6
*Plasmid 1 (pXAS220)*	219,634	62.8	230	0	0
*Plasmid 2(pXAS56)*	56,537	61.5	67	0	0
*Plasmid 3 (pXAS42)*	42,682	61.1	47	0	0
*Plasmid 4 (pXAS27)*	27,107	62.9	32	0	0
*Plasmid 5 (pXAS24)*	24,453	62.6	21	0	0
DAR72029					
Chromosome	5,178,793	64.9	4390	59	6
*Plasmid 1 (pXAS220)*	220,762	62.8	230	0	0
*Plasmid 2 (pXAS56)*	56,053	61.5	66	0	0
*Plasmid 3 (pXAS42)*	42,682	61.1	48	0	0
*Plasmid 4 (pXAS27)*	27,107	62.9	33	0	0
*Plasmid 5 (pXAS24)*	24,453	62.6	22	0	0
DAR73889					
Chromosome	5,222,784	64.8	4446	59	6
*Plasmid 1 (pXAS220)*	219,409	62.8	229	0	0
*Plasmid 2 (pXAS56)*	56,563	61.5	65	0	0
*Plasmid 3 (pXAS42)*	42,717	61.1	46	0	0
*Plasmid 4 (pXAS30)*	30,179	62.6	37	0	0
*Plasmid 5 (pXAS24)*	24,453	62.6	22	0	0
DAR73910					
Chromosome	5,326,504	64.8	4851	59	6
*Plasmid 1 (plasmid pF)*	116,539	63.3	142	0	0
*Plasmid 2 (pXAS38)*	38,592	62.2	38	0	0
*Plasmid 3 (pXAS28-2)*	28,174	63.0	37	0	0

**Table 3:** Counts of filtered TBLASTN hits of PthA1-4 in genomes sequenced in this study. Truncated proteins (truncTALE) are also presented with the location of the truncation (N-terminal or C-terminal).

Isolate	PthA1	Trunc-PthA1	PthA2	Trunc-PthA2	PthA3	Trunc-PthA3	PthA4	Trunc-PthA4
DAR73886	1	0	2	0	2	0	0	0
DAR84832	1	0	2	0	2	0	1	0
DAR72029	0	1^N^	1	1^N^,1^C^	1	1^N^	0	1^N^
DAR73889	1	1^N^	1	1^N^	1	1^N^	1	1^N^
DAR73909	0	1^N^	0	1^N^,1^C^	0	1^N^	0	1^N^
DAR73910	1	1^N^	1	1^N^	1	1^N^	1	1^N^

## Supplementary Materials

The following are available online at https://www.mdpi.com/2076-2607/8/12/1947/s1: Table S1: Table of National Center for Biotechnology Information (NCBI) isolates used in single-nucleotide polymorphism (SNP) and core genome phylogenies. Table S2: Table of type 3 secreted effectors. Table S3: Table of unique genes in A* genetic groups with functional annotation.
