# Supplementary material for: Comparative Genomics of Xanthomonas citri pv. citri A* Pathotype Reveals Three Distinct Clades with Varying Plasmid Distribution
Source: Microorganisms. 2020 Dec 8;8(12):1947. doi: 10.3390/microorganisms8121947 (PMC7764509; doi:10.3390/microorganisms8121947)
Supplement: Supplementary file 1 [file microorganisms-08-01947-s001.pdf]

microorganisms

MDPI

Table S1. Table of National Center for Biotechnology Information (NCBI) isolates used in single-nucleotide polymorphism (SNP) and core genome phylogenies.

Pathotype	Strain	BioSample	BioProject	Assembly	Level	Size (Mb)	Geographic Location
A	306	SAMN02603846	PRJNA297	GCA_000007165.1	Complete	5.27417	Brazil
A	5208	SAMN02911840	PRJNA255042	GCA_000961415.1	Complete	5.26922	USA
A	03-1638-1-1	SAMN07611881	PRJNA401937	GCA_002952295.1	Complete	5.49773	Argentina
A	A306	SAMN03278355	PRJNA193618	GCA_000816885.1	Complete	5.27417	Brazil
A*	AS270	SAMN02911852	PRJNA255042	GCA_000950845.1	Contig	5.27407	Saudi Arabia
A*	AS8	SAMN02911853	PRJNA255042	GCA_000950875.1	Contig	5.31182	Saudi Arabia
A*	AS9	SAMN02911854	PRJNA255042	GCA_000950855.1	Contig	5.30331	Saudi Arabia
A^W	Aw12879	SAMN02603165	PRJNA81931	GCA_000349225.1	Complete	5.39869	USA
A^W	AW13	SAMN02911848	PRJNA255042	GCA_000961435.1	Complete	5.39833	USA
A^W	AW14	SAMN02911849	PRJNA255042	GCA_000961455.1	Complete	5.39833	USA
A^W	AW15	SAMN02911850	PRJNA255042	GCA_000961475.1	Complete	5.39833	USA
A^W	AW16	SAMN02911851	PRJNA255042	GCA_000961495.1	Complete	5.39826	USA
A	BL18	SAMN02911845	PRJNA255042	GCA_000961395.1	Complete	5.2674	USA
A	FB19	SAMN02911846	PRJNA255042	GCA_000961375.1	Complete	5.26734	USA
A	FDC 1662	SAMN03317017	PRJNA273983	GCA_003122545.1	Scaffold	5.21728	Brazil
A*	FDC 1682	SAMN03317024	PRJNA273983	GCA_003122665.1	Scaffold	5.25271	Oman
A	FDC 628	SAMN03317028	PRJNA273983	GCA_003122605.1	Scaffold	5.17883	Brazil
A	FDC 636	SAMN03317019	PRJNA273983	GCA_003122575.1	Scaffold	5.1952	Brazil
A	FDC 654	SAMN03317029	PRJNA273983	GCA_003122625.1	Scaffold	5.21614	Brazil
A	FDC 828	SAMN03317018	PRJNA273983	GCA_003122565.1	Scaffold	5.1984	Brazil
A	gd2	SAMN02911834	PRJNA255042	GCA_000961355.1	Complete	5.22375	China
A	gd3	SAMN02911835	PRJNA255042	GCA_000961335.1	Complete	5.22375	China
A	JJ10-1	SAMEA2844848	PRJEB7180	GCA_000825185.2	Scaffold	5.18307	Mauritius
A*	JK143-11	SAMEA2844846	PRJEB7184	GCA_000825225.2	Scaffold	5.28418	Thailand
A*	JK143-9	SAMEA2827230	PRJEB7200	GCA_000825525.2	Scaffold	5.32065	Thailand
A	JK4-1	SAMEA2844844	PRJEB7186	GCA_000825265.2	Scaffold	5.211	China
A*	JK48	SAMEA2827235	PRJEB7195	GCA_000825425.2	Scaffold	5.3216	Saudi Arabia
A*	JM35-2	SAMEA2827561	PRJEB7189	GCA_000829945.2	Scaffold	5.25502	Saudi Arabia
A*	JS581	SAMEA2827560	PRJEB7190	GCA_000825325.2	Scaffold	5.2578	Iran
A*	JS582	SAMEA2827226	PRJEB7203	GCA_000825605.2	Scaffold	5.33892	Iran
A	jx4	SAMN02911836	PRJNA255042	GCA_000961315.1	Complete	5.2234	China
A	jx5	SAMN02911837	PRJNA255042	GCA_000961295.1	Complete	5.22342	China
A	jx-6	SAMN03765509	PRJNA286060	GCA_001028285.3	Complete	5.22335	China
A	LB100-1	SAMEA2844845	PRJEB7185	GCA_000825245.2	Scaffold	5.26656	Seychelles
A2	LE116-1	SAMEA2827228	PRJEB7201	GCA_000825545.2	Scaffold	5.43884	Mali
*	LE3-1	SAMEA2827236	PRJEB7194	GCA_000825405.2	Scaffold	5.36375	Ethiopia
A2	LG102	SAMEA2827232	PRJEB7198	GCA_000825485.2	Scaffold	5.39009	Bangladesh
A^W	LG115	SAMEA2827563	PRJEB7187	GCA_000825285.2	Scaffold	5.20845	India
A	LG117	SAMEA2827562	PRJEB7188	GCA_000825305.2	Scaffold	5.24968	Bangladesh
A2	LG97	SAMEA2827234	PRJEB7196	GCA_000825445.2	Scaffold	5.32388	Bangladesh
A	LG98	SAMEA2844847	PRJEB7183	GCA_000825205.2	Scaffold	5.17803	Bangladesh
A	LH201	SAMN05823148	PRJNA344031	GCA_001922105.1	Complete	5.46333	Reunion
A	LH276	SAMN05823145	PRJNA344031	GCA_001922065.1	Complete	5.49538	Reunion
A2	LH37-1	SAMEA2827558	PRJEB7192	GCA_000825365.2	Scaffold	5.40197	Senegal
A	LJ207-7	SAMN05823144	PRJNA344031	GCA_001922085.1	Complete	5.50554	Reunion
A	LL074-4	SAMN05823143	PRJNA344031	GCA_001922045.1	Complete	5.47167	Martinique
A	LM180	SAMN05823141	PRJNA344031	GCA_001939985.1	Chromosome	5.66709	Argentina
A	LM199	SAMN05822798	PRJNA344031	GCA_001939965.1	Chromosome	5.56881	Argentina
A	LMG 9322	SAMN02903175	PRJNA254373	GCA_001401655.1	Contig	5.10757	USA
A	LMG9322	SAMN05571463	PRJNA338819	GCA_002018575.1	Contig	5.14163	USA
A	mf20	SAMN02911847	PRJNA255042	GCA_000961275.1	Complete	5.2674	USA
A	MN10	SAMN02911841	PRJNA255042	GCA_000961255.1	Complete	5.22227	USA
A	MN11	SAMN02911842	PRJNA255042	GCA_000961235.1	Complete	5.22094	USA
A	MN12	SAMN02911843	PRJNA255042	GCA_000961215.1	Complete	5.22225	USA
A*	NCPPB 3607	SAMEA2827559	PRJEB7191	GCA_000825345.2	Scaffold	5.37498	India
A	NCPPB 3610	SAMEA2827231	PRJEB7199	GCA_000825505.2	Scaffold	5.26456	India
A2	NCPPB 3612	SAMEA2827237	PRJEB7193	GCA_000825385.2	Scaffold	5.34531	India
A2	NCPPB 3615	SAMEA2827227	PRJEB7202	GCA_000825565.2	Scaffold	5.43884	India
A*	NIGEB-386	SAMN03070127	PRJNA261284	GCA_001956275.1	Contig	5.33472	Iran
A*	NIGEB-88	SAMN03649471	PRJNA283400	GCA_002742455.1	Scaffold	5.3216	Iran

A	NT17	SAMN02911844	PRJNA255042	GCA_000961195.1	Complete	5.26925	USA
A^{W}	TX160042	SAMN06685652	PRJNA381640	GCA_002139975.1	Complete	5.50141	USA
A^{W}	TX160149	SAMN06685654	PRJNA381640	GCA_002139955.1	Complete	5.57542	USA
A^{W}	TX160197	SAMN06685696	PRJNA381640	GCA_002139995.1	Complete	5.51442	USA
A	UI6	SAMN02911838	PRJNA255042	GCA_000961175.1	Complete	5.22243	China
A	UI7	SAMN02911839	PRJNA255042	GCA_000961155.1	Complete	5.22229	China
A	Xcc29-1	SAMN07665076	PRJNA407058	GCA_003665475.1	Complete	5.27693	China
A	Xcc49	SAMN07638001	PRJNA407058	GCA_003665455.1	Complete	5.22085	China

Table S2. Table of type 3 secreted effectors.

Effector	Accession	Effector	Accession	Effector	Accession
AvrBs1	WP_168952546	XopAK	AAM38509	XopJ1	CAJ23833
AvrBs2	AAM34968	XopAL1	AAM40544	XopJ2-AvrBsT	ABM32744
AvrBs3-PthA1	AAM39226	XopAL2	CAP49952	XopJ3-AvrRxv	CAJ22102
AvrBs3-PthA2	AAM39243	XopAM	AAM40388	XopJ4-AvrXv4	CAD14528
AvrBs3-PthA3	AAM39261	XopAP	AAM37835	XopJ5	AAM42989
AvrBs3-PthA4	AAM39311	XopAQ	A0A0U5FGZ5_XANCI	XopK	AAM37930
AvrXccA2	AAM41674	XopAU	AAM36043	XopL	AAM37935
AvrXccA1	AAM43445	XopAV	AAM36044	XopM	AAM35309
HpaA	AAM35291	XopAW	AAM37794	XopN	AAM37631
HrpW	AAM37767	XopAY	AAM36044	XopO	CAJ22686
XoF2	AAM37630	XopAZ	AAM36229	XopP	AAM36080
XopA	AAM35307	XopB	CAJ22212	XopQ	AAM39163
XopAA	CAJ25516	XopC1	CAJ24112	XopR	AAM35169
XopAB	BAE69905	XopD	CAJ22068	XopS	AAM35207
XopAC	AAM41837	XopE1	AAM35178	XopT	ABJ89951
XopAD	AAM39048	XopE2	AAM39257	XopU	BAE69632
XopAE	AAM35284	XopE3	AAM38068	XopV	AAM35490
XopAF	A0A5C2EXZ7_9XANT	XopF1	CAJ22045	XopW	BAE66792
XopAG	AAM42870	XopF2	AAM37630	XopX	AAM35432
XopAH	AAM41397	XopG1	CAJ22929	XopY	BAE68243
XopAI	AAM38074	XopH1	CAJ19917	XopZ	AAM36871
XopAJ-AvrRxo1	CAJ26159	XopI	AAM35643		

Table S3. Table of unique genes in A* sub-pathotypes with functional annotation.

A*a	A*b	A*c
5,10-Methylenetetrahydrofolate reductase	Aerobic cobaltochelatase subunit CobS	2-Nitroimidazole transporter
Acetyl-/propionyl-coenzyme A carboxylase alpha chain	Alcohol dehydrogenase	3-Isopropylmalate dehydratase large subunit
ADP-ribosyl-[dinitrogen reductase] glycohydrolase	Alginate biosynthesis sensor protein KinB	3-Isopropylmalate dehydratase small subunit 1
Allophanate hydrolase	ATP-dependent DNA helicase PcrA	3-Keto-5-aminohexanoate cleavage enzyme
Antitoxin DinJ	ATP-dependent DNA helicase RecG	3-Methylmercaptopropionyl-CoA dehydrogenase
Blue-light-activated protein	ATP-dependent DNA helicase Rep	4-Hydroxybenzoate transporter PcaK
Coupling protein TraD	ATP-dependent RecD-like DNA helicase	4-Hydroxybenzoate--CoA/benzoate--CoA ligase
Cytochrome bd-II ubiquinol oxidase subunit 2	Beta sliding clamp	4-Hydroxybenzoyl-CoA thioesterase
D-alanyl-D-alanine carboxypeptidase	Cadmium, cobalt, and zinc/H(+)-K(+) antiporter	6-Hydroxy-3-succinoylpyridine 3-monooxygenase HspA
Methionine import system permease protein MetP	Cation efflux system protein CusA	Aromatic amino acid exporter YddG
Methyl-accepting chemotaxis protein IV	Cation efflux system protein CusB	Carbon storage regulator
Methylmalonate-semialdehyde dehydrogenase (acylating)	Chaperone protein HtpG	Chromosome partition protein Smc
mRNA interferase toxin YafQ	Chromosome partition protein Smc	Chromosome partition protein Smc
Multifunctional conjugation protein TraI	Chromosome partition protein Smc	Cobalt–zinc–cadmium resistance protein CzcA
Phospholipase D	Cobalt–zinc–cadmium resistance protein CzcA	DNA primase TraC
Protein TraI	Cobalt–zinc–cadmium resistance protein CzcB	DNA primase TraC
Protein translocase subunit SecA	Cobalt–zinc–cadmium resistance protein CzcC	DNA primase TraC
Protein virB8	Conjugal transfer protein TraG	DNA primase TraC
Putative endoribonuclease MazF	Copper chaperone CopZ	DNA topoisomerase 1
Putative endoribonuclease MazF	Copper resistance protein A	DNA topoisomerase 3
Putative endoribonuclease MazF	Copper-exporting P-type ATPase	DNA topoisomerase 3
Putative oxidoreductase YdgJ	Copper-transporting P-type ATPase	DNA translocase FtsK
Putative transposon Tn552 DNA-invertase bin3	Cytochrome bd-I ubiquinol oxidase subunit 1	DNA-binding protein HU
Response regulator receiver protein CpdR	DNA replication and repair protein RecF	Electron transfer flavoprotein subunit alpha
Ribosomal protein S12 methylthiotransferase RimO	DNA-binding protein HU	Electron transfer flavoprotein subunit beta
Type IV secretion system protein PtlE	DNA-invertase hin	Electron transfer flavoprotein-ubiquinone oxidoreductase
Type IV secretion system protein virB1	DNA-invertase hin	Extracellular phospholipase A1
Type IV secretion system protein virB1	ECF RNA polymerase sigma factor SigE	Gentisate 1,2-dioxygenase
Type IV secretion system protein virB1	Extracellular serine protease	Glutathione S-transferase GST-6.0
Type IV secretion system protein virB10	Formimidoylglutamase	Glutathione-independent formaldehyde dehydrogenase
Type IV secretion system protein virB10	Histidine ammonia-lyase	Glutathione-regulated potassium-efflux system ancillary protein KefF
Type IV secretion system protein VirB11	HTH-type transcriptional regulator HmrR	Glycyl-glycine endopeptidase ALE-1
Type IV secretion system protein VirB11	HTH-type transcriptional regulator HmrR	Group II intron-encoded protein LtrA
Type IV secretion system protein virB4	HTH-type transcriptional repressor YvoA	Group II intron-encoded protein LtrA
Type IV secretion system protein virB4	Iron-sulfur cluster carrier protein	Group II intron-encoded protein LtrA
Type IV secretion system protein virB5	Membrane-bound lytic murein transglycosylase C	Hca operon transcriptional activator HcaR
Type IV secretion system protein virB5	Modification methylase Eco57IB	Hca operon transcriptional activator HcaR

Type IV secretion system protein VirB6	mRNA interferase toxin HigB	HTH-type transcriptional regulator BetI
Type IV secretion system protein VirB6	Na(+)-translocating NADH-quinone reductase subunit F	HTH-type transcriptional regulator NimR
Type IV secretion system protein virB9	Plasmid replication initiator protein TrfA	HTH-type transcriptional regulator RcdA
Type IV secretion system protein virB9	Protein FecR	HTH-type transcriptional regulator SrpR
Vitamin B12 import ATP-binding protein BtuD	Protein TraC	HTH-type transcriptional repressor BepR
	Protein TraI	Insertion element IS6110 uncharacterized 12.0 kDa protein
	Protein translocase subunit SecA	Insertion element IS6110 uncharacterized 12.0 kDa protein
	Protein UmuC	L-carnitine dehydrogenase
	Protein UmuD	Long-chain-fatty-acid--CoA ligase
	Putative ATP-dependent helicase DinG	L-threonine 3-dehydrogenase
	Putative beta-lactamase HcpC	Membrane-bound lytic murein transglycosylase B
	Putative chromosome-partitioning protein ParB	Methylthioacryloyl-CoA hydratase
	Putative chromosome-partitioning protein ParB	Multidrug resistance protein MexA
	Putative signal peptide peptidase SppA	Multidrug resistance protein MexB
	Recombination-associated protein RdgC	NAD(P)H dehydrogenase (quinone)
	Ribosomal protein L11 methyltransferase	Osmo-dependent choline transporter BetT2
	Serine protease AprX	Outer membrane protein OprM
	Serine/threonine-protein kinase toxin HipA	Phenoxybenzoate dioxygenase subunit beta
	Single-stranded DNA-binding protein	Plasmid segregation protein ParM
	Single-stranded DNA-binding protein	Polyamine aminopropyltransferase
	Sporulation initiation inhibitor protein Soj	Prefoldin subunit alpha
	Thiol:disulfide interchange protein DsbC	Prophage integrase IntA
	Thiol:disulfide interchange protein DsbD	Prophage integrase IntS
	Toxin FitB	Protease HtpX
	Trans-acting regulatory protein HvrA	Protein PhlB
	tRNA(fMet)-specific endonuclease VapC	Protein TraI
	tRNA-Gly(gcc)	Protein translocase subunit SecA
	Type IIS restriction enzyme Eco57I	Protein translocase subunit SecA
	Type IV secretion system protein virB1	Putative glucose-6-phosphate 1-epimerase
	Tyrosine recombinase XerC	Putative lipoprotein YiaD
	Tyrosine recombinase XerC	Putative TonB-dependent receptor BfrD
	Urocanate hydratase	Psutative trans-3-hydroxy-L-proline dehydratase
	Vitamin B12 transporter BtuB	Putative xanthine dehydrogenase YagR molybdenum-binding subunit
	Vitamin B12 transporter BtuB	Putative xanthine dehydrogenase YagR molybdenum-binding subunit
	Vitamin B12 transporter BtuB	Recombination-associated protein RdgC
		Silver exporting P-type ATPase
		Single-stranded DNA-binding protein
		Single-stranded DNA-binding protein
		Sphingomyelinase
		Sporulation initiation inhibitor protein Soj

	Threonine/homoserine exporter RhtA
	Toluene-4-sulfonate monooxygenase system iron-sulfur subunit TsaM1
	Transcriptional regulator SlyA
	Twitching mobility protein
	Tyrosine recombinase XerC
	Tyrosine recombinase XerD
	Ureidoglycolate lyase
	Xylose isomerase
